# Adolescent Idiopathic Scoliosis and Pregnancy

**DOI:** 10.7759/cureus.46782

**Published:** 2023-10-10

**Authors:** Khizar R Nandoliya, Nishanth S Sadagopan, Amr Alwakeal, Hanna Kemeny, Michael Cloney, Nader S Dahdaleh, Tyler Koski, Najib El Tecle

**Affiliations:** 1 Neurological Surgery, Northwestern University Feinberg School of Medicine, Chicago, USA

**Keywords:** scoliosis progression, pregnancy, neuraxial anesthesia, idiopathic scoliosis, caesarean section, back pain

## Abstract

Objective

This study examined the interaction between adolescent idiopathic scoliosis (AIS) and pregnancy, focusing on pregnancy outcomes, changes in back pain, and anesthesia use.

Methods

A retrospective analysis was conducted on adult patients with AIS who gave birth at our institution between 2006 and 2022.

Results

A total of 163 AIS patients with 263 pregnancies were included. The median age at delivery was 33 (range 18 to 50) years. Among 157 patients with information on prior scoliosis treatment, 66.9% had not received treatment, 20.4% had undergone spinal fusion, and 12.7% had received bracing. Of the 260 pregnancies with available data, 90.4% were delivered at term and 8.5% were preterm. Of the 257 pregnancies with information on anesthesia type, 35.0% received epidural anesthesia, 17.9% received spinal anesthesia, 37.7% received combined spinal and epidural anesthesia, 8.2% received no anesthesia, and 1.2% received intravenous or general anesthesia. Difficulty administering neuraxial anesthesia was reported in 6.1% of cases, and these patients were less likely to receive combined spinal and epidural anesthesia (6.3% versus 39.8%, p = 0.0123). Among 116 cases with recorded back pain during pregnancy, 67.2% reported increased pain, 31.9% reported similar pain, and one patient reported decreased pain. Of the 16 patients with pre and postpartum radiographs, eight showed a Cobb angle increase ≥ 3°, with five patients having an increase ≥ 5°.

Conclusions

Pregnancy can exacerbate back pain and pose challenges for neuraxial anesthesia in some AIS patients. Further large-scale, multi-institutional studies with standardized data collection are needed to fully understand the impact of pregnancy on AIS.

## Introduction

Adolescent idiopathic scoliosis (AIS), defined by its onset in children between 10 and 18 years old, is the most common spinal deformity in adolescents and young adults [1]. It has a much higher incidence and greater severity in females than males [1-5]. Thus, during pregnancy, worsening back pain and difficulty administering spinal or epidural anesthesia during delivery are salient concerns for many patients with AIS.

The existing literature investigating the relationship between AIS and pregnancy is sparse and has yielded conflicting results. The objective of this study is to characterize how AIS and pregnancy influence each other by describing changes in scoliotic spinal curvature, changes in back pain during pregnancy and after delivery, and anesthesia usage and problems that may arise with neuraxial anesthesia administration during delivery.

## Materials and methods

This study was conducted in accordance with the Health Insurance Portability and Accountability Act (HIPAA) guidelines. We performed a retrospective review of all adult (age 18 and above) patients with a history of AIS who gave birth at our institution between September 2006 and March 2023. Patients with non-idiopathic scoliosis and infantile or juvenile idiopathic scoliosis were excluded.

Data collected included patient age at delivery, spinal levels affected by AIS, pre- and post-partum Cobb angle, pregnancy outcomes, delivery method (vaginal or caesarean section), type of anesthesia used, and whether any difficulty administering anesthesia was recorded. Spinal levels were classified as thoracic (T2-T12), lumbar (L1-L4), or thoracolumbar. Pre- and post-partum Cobb angles were only calculated for patients with pre- and post-partum radiographs available in the electronic medical record. Cobb angles were calculated in Visage (Visage Imaging GmbH, Berlin, Germany) by authors K.R.N. and N.E.T. independently. Pregnancy outcomes were classified based on estimated gestational age (EGA) at the time of delivery as term (EGA 37 weeks and zero days), preterm (EGA < 37 weeks), miscarriage (pregnancy loss before the 20th week), or stillbirth (pregnancy loss at or after 20 weeks EGA). Prepartum spinal curvature was considered to have worsened if the Cobb angle increased by at least 3° in pre and postpartum radiographs.

Fisher’s exact tests were reported for categorical variables. Medians and Wilcox rank sum tests were reported for continuous variables. The decision to report medians rather than means was based on the results of Shapiro-Wilks tests, suggesting non-normal data. A p-value less than 0.05 was considered statistically significant for all analyses. All statistical analyses were performed in RStudio version 4.2.0 (Posit PBC, Boston, MA, USA).

## Results

A total of 163 AIS patients with 263 pregnancies were identified. The median (range) age at the time of delivery was 33 (18 to 50) years. Out of 260 pregnancies with available data on outcomes, 235 (90.4%) were at term, 22 (8.5%) were preterm, two resulted in miscarriage, and one resulted in stillbirth. Of these 263 pregnancies, 178 (68.5%) were delivered vaginally and 82 (31.5%) via c-section. Out of 128 patients with information on the spinal level of scoliosis, 47 (36.7%) had thoracic scoliosis, 34 (26.6%) had lumbar scoliosis, and 47 (36.7%) had thoracolumbar scoliosis. The incidence of c-section was 29.0%, 22.8%, and 34.2% for pregnancies in which the patient had thoracic, lumbar, and thoracolumbar scoliosis, respectively (p = 0.3704). Out of 157 patients with available data on prior treatment for scoliosis, 105 (66.9%) had not received any treatment, 32 (20.4%) had received spinal fusion, and 20 (12.7%) had received back bracing. The incidence of c-section was 36.9% for pregnancies in which patients had received spinal fusion and 28.2% for patients who had not received any treatment (p = 0.8643). Patient and clinical characteristics are summarized in Table 1.

**Table 1:** Information on patient demographics, pregnancy outcomes, delivery methods, and prior treatments for scoliosis

Demographics	
Total number of deliveries	263
Median age in years (range)	33 (18 – 50)
Pregnancy Outcomes	n = 260 deliveries
Term	235 (90.4%)
Preterm	22 (8.5%)
Miscarriage	2 (0.01%)
Stillbirth	1 (0.004%)
Delivery Method	n = 260 deliveries
Vaginal	178 (68.5%)
C-section	82 (31.5%)
Affected Spinal Level	n = 128 patients
Thoracic	47 (36.7%)
Lumbar	34 (26.6%)
Thoracolumbar	47 (36.7%)
Prior Scoliosis Treatments	n = 157 patients
None	105 (66.9%)
Spinal fusion	32 (20.4%)
Brace	20 (12.7%)
Anesthesia Type	n = 257 deliveries
Epidural	90 (35.0%)
Spinal	46 (17.9%)
Combined spinal/epidural	97 (37.7%)
None	21 (8.2%)
Intravenous or general	3 (1.2%)
Pain Levels	n = 116 patients
Increase	78 (67.2%)
No change	37 (31.9%)
Decrease	1 (0.01%)

Administration of anesthesia during labor in patients with AIS

Information on anesthesia use was available for 257 pregnancies. Of these, 90 (35.0%) received epidural anesthesia, 46 (17.9%) received spinal anesthesia, 97 (37.7%) received combined spinal and epidural anesthesia, 21 (8.2%) received no anesthesia, and 3 (1.2%) received intravenous or general anesthesia. Difficulty administering anesthesia was reported in 16 (6.1%) pregnancies. Patients who faced difficulty in being administered anesthesia were less likely to receive combined spinal and epidural anesthesia than patients who did not experience difficulty (6.3% versus 39.8%, p = 0.0123), but there were no differences in delivery method or prior treatment for AIS (Table 2).

**Table 2:** Anesthesia type, delivery method, and history of prior scoliosis treatment in patients with and without difficulty receiving anesthesia * Information on prior treatment was available for 233 out of 241 patients

Variables	No difficulty with anesthesia, n = 241	Difficulty with anesthesia, n = 16	p-value
Anesthesia type			0.0123
Epidural	81 (33.6%)	9 (56.3%)	
Spinal	42 (17.4%)	4 (25.0%)	
Combined spinal/epidural	96 (39.8%)	1 (6.3%)	
None	20 (8.3%)	1 (6.3%)	
Intravenous	2 (0.01%)	1 (6.3%)	
Delivery method			0.0567
Vaginal	168 (69.7%)	10 (62.5%)	
Caesarean section	73 (30.3%)	6 (37.5%)	
Prior Treatment*			0.7797
None	153/233 (65.7%)	10/16 (62.5%)	
Fusion	53/233 (22.7%)	3/16 (18.8%)	
Brace	27/233 (11.6%)	3/16 (18.8%)	

In four patients, anesthesia placement was attempted at multiple spinal levels before it was successfully achieved. In another five patients, administration of anesthesia was achieved, but patients reported unilateral or otherwise incomplete anesthesia. In five patients, multiple unsuccessful attempts were noted without further details. In one patient, hip pain was reported during the threading of the catheter, which did not resolve for the duration of labor. An accidental dural puncture was reported in the final patient.

Changes in back pain and spinal curvature

Information on levels of back pain during or after pregnancy was explicitly recorded for 116 pregnancies. An increase in back pain was reported in 78 (67.2%) pregnancies, no change in 37 (31.9%), and a decrease during one pregnancy. The median maternal age for pregnancies in which back pain increased was 33.0 (IQR 30.0-35.8) years, compared to 33.0 (IQR 31.3-35.0) years in pregnancies in which pain did not increase (p = 0.4899). Difficulty administering anesthesia was encountered in three (8.1%) pregnancies in which patients experienced increased back pain, compared to nine (11.5%) pregnancies in which patients did not experience increased back pain (p = 0.7484).

**Figure 1:**
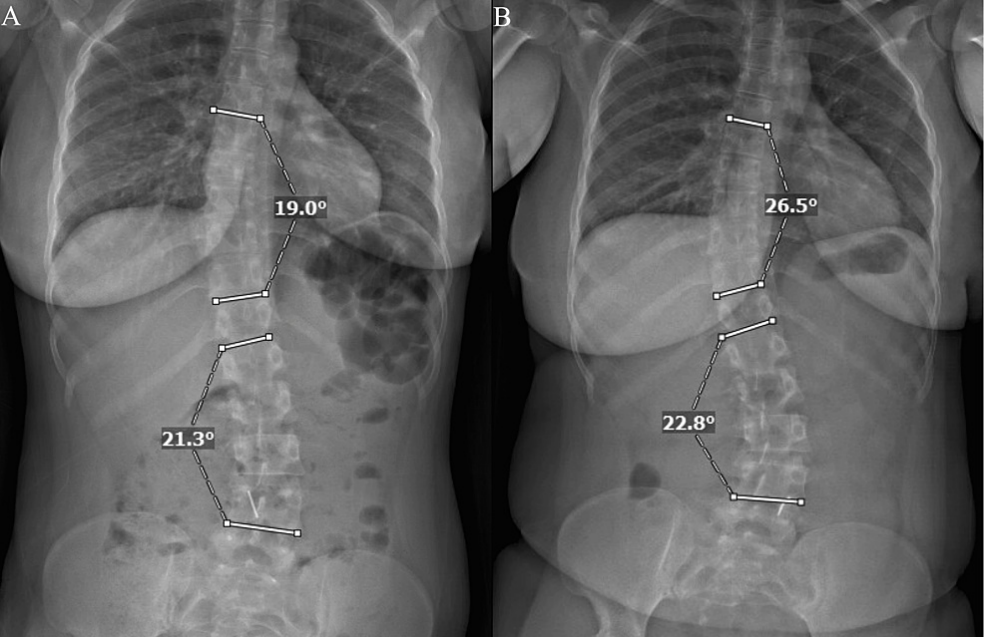
Cobb angles in patient 11 A: Before pregnancy, B: After pregnancy

Pre and postpartum radiographs were available for 16 patients (Table 3, Figure 1). The median time between first delivery and postpartum radiograph was 43 (IQR 17-63.5) months. Six (37.5%) patients had one delivery between radiographs, eight (50%) had two deliveries, and two (12.5%) had three deliveries. Nine (56.3%) patients had scoliosis of the thoracic section alone, six (37.5%) had scoliosis of both the thoracic and lumbar sections, and one patient (6.3%) had lumbar scoliosis alone. Eight (50.0%) patients had progression of scoliotic curvature between the pre and postpartum radiographs. Of these, five had a change in curvature of at least 5°, and the greatest change was 8°. The median (range) Cobb angle increased from 29° (9° to 57°) prepartum to 30° (10° to 66°) postpartum (p = 0.0017).

**Table 3:** Pre and postpartum Cobb angle in patients with available pre and postpartum radiographs * The median (range) prepartum Cobb angle was 29° (9° to 57°), compared to 30° (10° to 66°) postpartum (p = 0.0017). ** Curve changes were classified as stable if the Cobb angle increased < 3° or worse if the Cobb angle increased ≥ 3°

Patient	Spinal levels affected	Deliveries between X-rays	Months between first delivery and postpartum X-ray	Prepartum Cobb angle	Postpartum Cobb angle	Change in Cobb angle*	Curve change**	Prior treatment
1	Thoracic	1	9	20°	22°	2°	Stable	Unknown
2	Thoracic, Lumbar	2	12	38°, 44°	39°, 44°	1°, 0°	Stable	Brace
3	Thoracic, Lumbar	1	15	27°, 32°	28°, 37°	1°, 5°	Stable, Worse	None
4	Thoracic	1	15	41°	49°	8°	Worse	Brace
5	Thoracic, Lumbar	1	19	26°, 57°	26°, 66°	0°, 9°	Stable, Worse	Fusion
6	Thoracic	3	32	34°	38°	4°	Worse	None
7	Lumbar	2	41	27°	33°	6°	Worse	None
8	Thoracic	2	42	25°	27°	2°	Stable	Brace
9	Thoracic, Lumbar	2	44	12°, 9°	15°, 10°	3°, 1°	Worse, Stable	None
10	Thoracic, Lumbar	2	45	29°, 30°	29°, 30°	0°, 0°	Stable, Stable	Fusion
11	Thoracic, Lumbar	2	48	19°, 21°	27°, 23°	8°, 2°	Worse, Stable	None
12	Thoracic	2	61	49°	50°	1°	Stable	Brace
13	Thoracic	1	66	23°	25°	2°	Stable	Fusion
14	Thoracic	1	83	52°	54°	2°	Stable	Fusion
15	Thoracic	2	84	58°	58°	0°	Stable	None
16	Thoracic	3	86	25°	28°	3°	Worse	None

## Discussion

Key results

We found that difficulty administering anesthesia was reported in 6% of our cohort. Patients in whom difficulty administering anesthesia was encountered were less likely to receive combined spinal and epidural anesthesia, but there were no differences in delivery methods or prior scoliosis treatment. Furthermore, nearly 70% of pregnancies in our cohort of AIS patients were associated with an increase in back pain. In a limited subgroup of patients with pre and postpartum radiographs, 50% had worsening scoliotic curvature after pregnancy.

Interpretation

Our cohort had an 8.5% incidence of preterm births, relatively similar to the national incidence of 10.1% at the time of writing [6]. In comparison, a cohort of 84 AIS patients published by Chan et al. reported a preterm birth rate of 21.4% [7]. Chan et al. wrote that this finding may be due to a higher number of patients seeking specialized care at their tertiary care center than because of AIS itself [7]. The findings in our cohort, in which the incidence of preterm births has not increased, support this assertion. The incidence of c-section in our cohort (31.5%) was comparable to the incidence in our state (31.2%) [6]. In comparison, Grabala et al. found that women who had undergone scoliosis surgery were twice as likely to need c-sections as healthy controls, and that as the lowest instrumented vertebra moved caudal to L4, the frequency of c-sections increased [8]. In contrast, a separate study by Swany et al. found that fusion caudal to L3 was not associated with a greater incidence of c-section [9]. In our study, the incidence of c-section was not associated with the spinal level of scoliosis or fusion. Together, these data suggest that there continues to be controversy over the impact of AIS on pregnancy outcomes, and there is a need for larger, prospective studies with greater statistical power. 

In our cohort, anesthesia was successfully administered in 94% of patients. In comparison, a prior study of 19,259 deliveries reported an adequate analgesia rate of 98.8% [10]. Patients who faced difficulty receiving anesthesia were less likely to receive combined spinal and epidural anesthesia, which has been shown to have superior analgesic effects and lower complication rates than epidural anesthesia alone [11]. Reports of successful neuraxial anesthesia administration in AIS patients during delivery range widely. Chan et al. found that successful neuraxial anesthesia was administered in 99% of patients in whom it was attempted [7], whereas Cao et al. reported that only 3% of AIS patients in their cohort received neuraxial anesthesia [12]. At least some of this heterogeneity likely derives from differences in sample sizes, as the cohort published by Cao et al. only included 11 AIS patients. Furthermore, a 2023 matched cohort study by Chapek et al., including 1810 females with idiopathic scoliosis, found that patients treated with spinal fusion had a 38% decreased relative risk of receiving epidural anesthesia compared to patients treated nonoperatively [13], which suggests that differences in scoliosis management and curve severity may further contribute to the heterogeneity. Such heterogeneity makes it difficult to extrapolate the findings of individual cohorts to the wider AIS population. A potential area of future studies is to create multidisciplinary and multi-institutional standardized documentation protocols for pregnant patients with scoliosis so that analgesia outcomes can be objectively assessed with the ultimate goal of informing patient education and care delivery.

Other important areas of concern for pregnant AIS patients are back pain and changes in curvature. In our cohort, 67% of patients reported subjective increases in back pain during pregnancy or after delivery. In comparison, a prospective study of 855 Swedish pregnant women reported a 27% six-month incidence of back pain [14]. In pregnant AIS patients, reported rates of increased back pain range from 17% to 77% [15-19]. According to a systematic review by Dewan et al., which incorporated more than 3125 AIS patients, pregnancy-related back pain in most AIS patients is non-debilitating and resolves after delivery [20]. However, in a report of 146 patients treated with Harrington rods, Orvomaa et al. reported that more than 10% requested sick leave during their pregnancy due to back pain [21]. Falick-Michaeli et al. reported a small cohort of 17 AIS patients who required hospitalization for severe back pain, with pain persisting beyond pregnancy in 76% of patients [18]. Thus, although most literature suggests pregnancy-related back pain in AIS patients is not usually debilitating or permanent, patients and clinicians should be proactive in its management to protect quality of life.

Changes in spinal curvature after pregnancy were not uncommon in our cohort. Although nearly all our patients experienced some increase in Cobb angle, only about 50% of patients with available pre and postpartum radiographs had a Cobb angle change of ≥ 3°. Our findings are in agreement with prior studies, which found a mild to moderate increase in spinal curvature after pregnancy in some AIS patients [15,17,21-25]. For example, Danielsson et al. reported a mean progression of 3.5° ± 3.9° in a large cohort of 234 patients with pre and postpartum radiographs [18]. However, Chan et al. did not find significant changes in pre and postpartum Cobb angles, which they attributed to a small sample size [7]. As obtaining routine scoliosis radiographs into adulthood is not standard of care at most institutions, the extrapolation of data related to Cobb angle changes is severely hampered by underpowered sample sizes. Prospective studies in which patients receive pre and postpartum scoliosis radiographs represent a potential area of future study to better elucidate the clinical significance of pregnancy-related changes in spinal curvature.

Limitations

We acknowledge several limitations of this study. First, this is a retrospective review of a single institution’s electronic medical record. Thus, the availability of data was limited to the patient characteristics and outcomes available therein. The reporting of scoliosis-related anesthesia complications in the electronic medical record was often incomplete, which limited our ability to further investigate the specific reasons for such complications. Additionally, we relied on patient-reported comparisons in the medical record of their pain levels before and after pregnancy, and details on interventions for intrapartum or postpartum back pain were rarely recorded. Such data are subject to selection bias, as patients who experience greater pain may be more likely to discuss it with clinicians compared to patients with unchanged pain levels. Furthermore, this study lacks a control group of non-AIS pregnant patients. To provide this critically important context, we discuss the incidences of delivery methods, pregnancy results, successful neuraxial anesthesia, and back pain in our cohort of AIS patients compared to those found in the literature on the general population. Lastly, our examination of changes in pre and postpartum curvature was limited by the availability of pre and postpartum radiographs. Furthermore, due to the lack of radiographs immediately prior to pregnancy, it is difficult to precisely determine whether a change in curvature happened before or during pregnancy. Due to their retrospective nature, this has been a consistent limitation of studies examining changes in scoliotic curvature after childbearing [7,15,17,24]. Future studies should be conducted prospectively in larger, multi-institutional cohorts to eliminate such limitations.

## Conclusions

Patients with AIS had comparable rates of c-section to the general population, and even among patients with AIS, a history of spinal fusion was not associated with an increased incidence of c-section. Adolescent idiopathic scoliosis may be associated with difficulty administering anesthesia in a minority of patients, which can lead to a lower rate of combined spinal and epidural anesthesia usage. Furthermore, most patients with AIS will experience increased back pain during and after pregnancy. Changes in Cobb angle are seen in many patients, but the clinical significance of these changes remains unknown. Larger, prospective studies are needed to better investigate these outcomes and guide patient education.
